# Identification of an unique CXCR4 epitope whose ligation inhibits infection by both CXCR4 and CCR5 tropic human immunodeficiency type-I viruses

**DOI:** 10.1186/1742-4690-8-84

**Published:** 2011-10-22

**Authors:** Tetsuya Adachi, Reiko Tanaka, Akira Kodama, Mineki Saito, Yoshiaki Takahashi, Aftab A Ansari, Yuetsu Tanaka

**Affiliations:** 1 Department of Immunology, Graduate School of Medicine, University of the Ryukyus, Okinawa, Japan; 2 Department of Pathology, Emory University School of Medicine, Atlanta, GA 30322, USA

## Abstract

**Background:**

Small chemical compounds which target chemokine receptors have been developed against human immunodeficiency virus type 1 (HIV-1) and are under investigation for use as anti-HIV-1 microbicides. In addition, monoclonal antibodies (mAbs) against chemokine receptors have also been shown to have anti-HIV-1 activities. The objective of the present study was to screen a panel of three anti-CXCR4 specific monoclonal antibodies (mAbs) for their ability to block the HIV-1 infection using *in vitro *activated primary peripheral blood mononuclear cells (PBMCs).

**Results:**

PBMCs from normal donors were pre-activated with anti-CD3 and anti-CD28 mAbs for 1 day, and aliquots were infected with a low dose of CCR5-tropic (R5), CXCR4 tropic (X4) or dual tropic (X4R5) HIV-1 isolates and cultured in the presence of a panel of anti-CXCR4 mAbs. The panel included clones A145 mAb against the N-terminus, A120 mAb against a conformational epitope consisting of extracellular loops (ECL)1 and ECL2, and A80 mAb against ECL3 of CXCR4. Among these mAbs, the A120 mAb showed the most potent inhibition of infection, by not only X4 but surprisingly also R5 and X4R5 HIV-1. The inhibition of R5 HIV-1 was postulated to result from the novel ability of the A120 mAb to induce the levels of the CCR5-binding β-chemokines MIP-1α, MIP-1β and/or RANTES, and the down modulation of CCR5 expression on activated CD4^+ ^T cells. Neutralizing anti-MIP-1α mAb significantly reversed the inhibitory effect of the A120 mAb on R5 HIV-1 infection.

**Conclusions:**

The **d**ata described herein have identified a unique epitope of CXCR4 whose ligation not only directly inhibits X4 HIV-1, but also indirectly inhibits R5 HIV-1 infection by inducing higher levels of natural CCR5 ligands.

## Background

CXCR4 and CCR5 belonging to the family of G-protein coupled receptors (GPCR) serve as receptors for the CXC-chemokine stromal derived factor 1 (SDF-1) and the CC-chemokines MIP-1α, MIP-1β and RANTES, respectively. The ligation of these chemokine receptors transmits a number of intracellular signals, and the receptors also serve as co-receptors for HIV-1 [1-5]. Under normal physiological conditions, CXCR4 molecules form closely linked dimers [6] and heterodimers with other chemokine receptors including CCR5 [7]. CXCR4 is expressed extracellularly, consisting of an N-terminal (NT) region and extracellular loops (ECL) 1, ECL2 and ECL3. Several lines of evidence indicate that the interaction between CXCR4 and SDF-1 or HIV-1 involves multiple domains of the receptor. For example, while the NT and the ECL2 domains appear to be critical for SDF-1 binding and signaling, the regions contiguous to the ECL2 and ECL3 have been implicated in HIV-1 co-receptor activity and homologous cell adhesion [8-11]. Studies with CXCR4 mutants have revealed that the HIV-1 co-receptor activity of CXCR4 is independent of its ability to function as a chemokine receptor and/or transduce intracellular signaling [11,12].

Current and prospective anti-HIV-1 therapy includes the use of small chemical compounds which target chemokine receptors that are termed viral occupancy inhibitors (VIROC) [13]. In addition, mAbs against chemokine receptors have also been shown to have a potential for HIV-1 inhibition. For example, an anti-human CCR2 mAb that is neither an agonist nor an antagonist blocks both X4 and R5 HIV-1, due to oligomerization of CCR2 with CCR5 and CXCR4, but not receptor down-modulation [14]. In addition, an unique mAb with specificity for the N-terminus region of CCR5 that does not block the interaction between HIV-1 gp120 and CCR5, blocks R5 HIV-1 infection by inducing CCR5 dimerization [15].

Herein, we examined a series of three rat IgG anti-human CXCR4 mAbs made by our laboratory [16], and we demonstrate that clone A120, that recognizes a conformational epitope encompassing the ECL1 and ECL2 domains of CXCR4, has a unique functional property. Thus, the interaction of the A120 mAb with CXCR4 inhibits not only X4, but also R5 HIV-1 infection of in vitro activated PBMCs, via mechanisms detailed herein. The novel anti-CXCR4 mAb function described in this study potentially provides a unique adjunct to conventional anti-HIV-1 chemotherapy with activity against not only CXCR4 but also CCR5 and dual tropic HIV-1.

## Results

### Suppressive effects of anti-CXCR4 mAbs on HIV-1 infection in primary activated PBMCs

**Figure 1:**
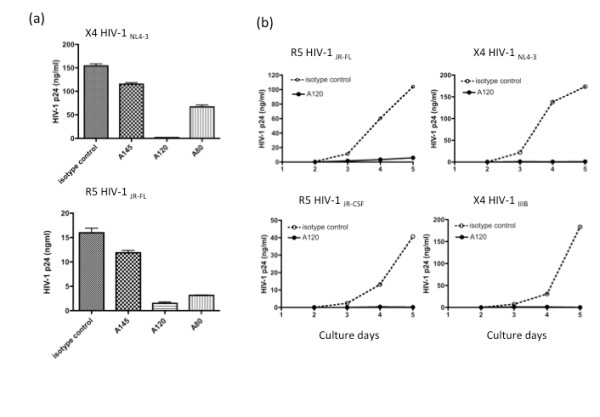
**Inhibition of HIV-1 infection in activated PBMCs by anti-CXCR4 mAbs**. (a) PBMCs activated with anti-CD3/CD28 for 1 day were infected with either R5 HIV-1_JR-FL _or X4 HIV-1_NL4-3 _for 2 hours, washed and then cultured in the presence of 10 μg/ml of the A145, A120, A80 rat IgG mAbs or isotype control rat IgG mAb mixture. After 5 days, virus production in the culture supernatants was determined by p24 ELISA. (b) Activated PBMCs infected with R5 HIV-1_JR-FL_, R5 HIV-1_JR-CSF_, X4 HIV-1_NL4-3 _or X4 HIV-1_IIIB _were aliquoted and cultured in the presence of 10 μg/ml of the A120 mAb or isotype control mAb. The p24 levels in the culture supernatants were monitored daily by ELISA. Data shown for both (a) and (b) are representative of 3 independent experiments using PBMCs from different donors.

We first tested our 3 different anti-CXCR4 mAb clones (A145, A120 and A80) for their potential to inhibit the infection of the prototype X4 HIV-1_NL4-3 _and for purposes of controlling the prototype R5 HIV-1_JR-FL _in *in vitro *activated primary PBMC cultures. None of these anti-human CXCR4 mAbs cross-reacts with human CCR5, and only the A120 mAb can block the SDF-1-mediated Ca^2+ ^influx [16]. Thus, the PBMCs infected with low levels of HIV-1 (at a multiplicity of infection of lower than 0.01) were cultured for 5 days in the presence or absence of 10 μg/ml of either anti-CXCR4 mAb or isotype control. As shown in Figure 1a, while the A145 mAb had minimal inhibitory effect, the A120 and A80 mAbs markedly inhibited the infection of the X4, but to our surprise, also the R5 HIV-1 strain. Since the inhibitory potential of the A120 mAb was the highest among these mAbs, we selected the A120 mAb for further characterization. Although the production of HIV-1 from activated PBMCs was influenced by culture conditions, mostly cell concentration at time of infection and cultivation steps, as shown in Figure 1b, the inhibitory effect of A120 mAb was further confirmed using an additional R5 (JR-CSF) and X4 (IIIB) HIV-1 strains.

**Figure 2:**
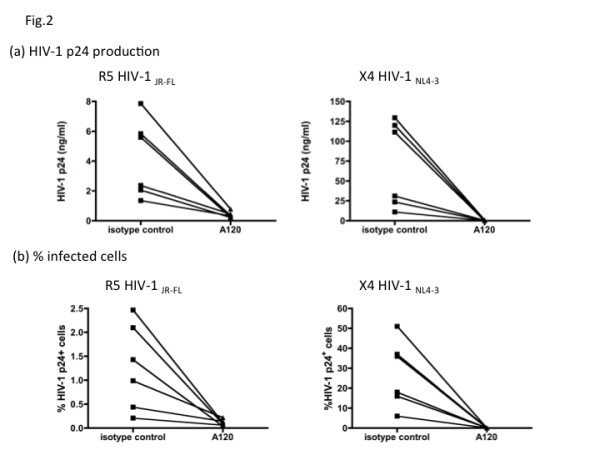
**The A120 mAb-mediated inhibition of HIV-1 infection in activated PBMCs from different donors**. Activated PBMCs from 6 different donors were infected with either R5 HIV-1_JR-FL _or X4 HIV-1_NL4-3 _for 2 hours. After extensive washing, the PBMCs were aliquoted and cultured in the presence of A120 or isotype control IgG at 10 μg/ml. (a) After 3~5 days, virus production was determined by p24 ELISA in the culture supernatants, and values obtained on day 4 are shown as representative. P values were 0.007 and 0.032 for R5 HIV-1 and X4 HIV-1, respectively. (b) The PBMC samples obtained on day 4 after infection were fixed and permeabilized, and then stained with anti-HIV-1 p24 mAb labeled with Alexa Fluor 488 and examined by flow cytometry. The frequencies (percentages) of p24^+ ^cells were plotted. P values were 0.026 and 0.031 for R5 HIV-1 and X4 HIV-1, respectively. Representative data from 3 independent experiments are shown.

To examine tPBMC donor variabilities, the ability of the A120 mAb to inhibit R5 HIV-1_JR-FL _and X4 HIV-1_NL4-3 _in activated PBMCs from 6 different unrelated donors was also studied. Viral production was quantitated by measuring both the levels of p24 and the frequency of infected cells using flow cytometry as outlined in the methods section. As seen in Figure 2a, whereas there was indeed considerable variability in the relative susceptibility of *in vitro *activated PBMCs from different donors to support R5 and X4 HIV-1 infection, the addition of the A120 mAb to the cultures showed variable levels of moderate to significant inhibition in each case (differences in the ability of PBMCs from different donors to support R5 versus X4 HIV-1 is an interesting subject that is currently under study). In addition, the fact that the addition of the A120 mAb also inhibited the increase in the frequency of infected cells as determined by flow cytometry (Figure 2b) suggests that the A120 mAb inhibits new infection in the cultures. To our knowledge, this is the first report of an anti-CXCR4 mAb that inhibits infection of both X4 and R5 HIV-1 strains in activated PBMCs.

**Figure 3:**
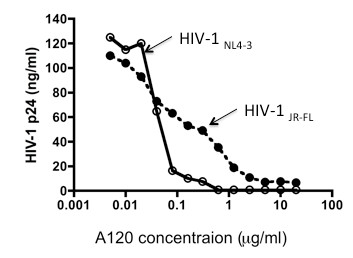
**Dose responses of the A120 mAb-mediated inhibition of R5 and X4 HIV-1 infection in activated PBMCs**. Activated PBMCs from the donors were infected with either R5 HIV-1_JR-FL _or X4 HIV-1_NL4-3_. After washing, the PBMCs were aliquoted and cultured in the presence of graded concentrations of the A120 mAb for 4 days. Virus production in the culture supernatant was determined by p24 ELISA. Representative data from 3 independent experiments using 3 different donors' PBMCs are shown.

**Figure 4:**
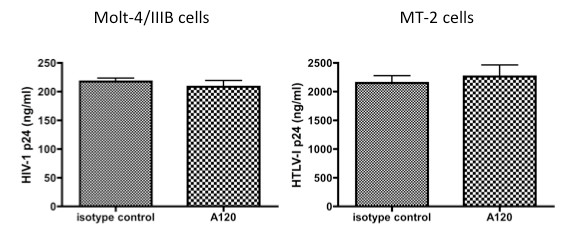
**The A120 mAb does not affect HIV-1 and HTLV-I production from producer cell lines**. The X4 HIV-1_IIIB _producer cell line (Molt-4/IIIB) and the HTLV-I producer cell line (MT-2) cells were cultured in the presence of 10 μg/ml of A120 or control mAb for 3 days. The culture supernatants were assayed for HIV-1 p24 and HTLV-I p24 by standard ELISA.

Dose response studies were conducted next to determine whether differences exist in the inhibition of R5 as compared with X4 HIV-1. As seen in Figure 3, maximum inhibition was achieved at a concentration of more than 5 μg/ml and 0.6 μg/ml for R5 and X4 HIV-1, respectively. The difference noted in the titration curves indicates that the potential mechanisms for A120 mAb-mediated R5 and X4 HIV-1 suppression are likely to be distinct from each other. The inhibition of virus replication by the addition of the A120 mAb in these cultures was not secondary to the presence of non-specific inhibitors in the A120 mAb preparation since the addition of the same A120 mAb preparation to the CXCR4 expressing HIV-1 producing Molt-4/IIIB cell line and the HTLV-1 producing MT-2 cell line had no detectable effect on virus production (Figure 4). Because the two cell lines express high levels of CXCR4 that readily binds the A120 mAb, it appears that the mere ligation of CXCR4 via A120 mAb epitope does not interfere with the virus production from these cell lines.

**Figure 5:**
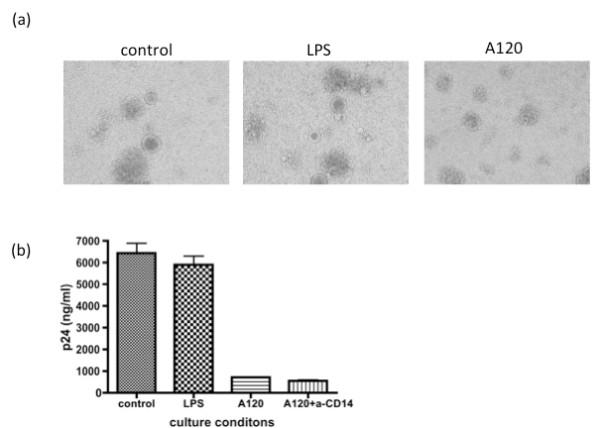
**LPS is not involved in the A120 mAb-mediated inhibition of HIV-1 infection**. Activated PBMCs infected with R5 HIV-1_JR-FL _were cultured in the presence or absence of LPS (0.1 μg/ml) or the A120 mAb with or without anti-CD14 mAb. After 4 days, syncytium formation and virus production in the culture supernatants were determined microscopically (a) and using a p24 ELISA kit (b), respectively.

One of the trivial explanations for the R5 HIV-1 suppression by the anti-CXCR4 mAb could be ascribed to the potential presence of LPS in the A120 mAb preparation. However, it is highly unlikely, because (1) the A120 mAb preparation contained little LPS since it was repeatedly passed through a polymyxin B column to eliminate possible LPS contamination, (2) exogenously added LPS at 0.1 μg/ml did not inhibit R5 HIV-1 infection in the same culture conditions, and (3) the inclusion of anti-human CD14 mAb that blocks the binding of LPS failed to interfere with the A120 mAb-mediated R5 HIV-1 inhibition (Figure 5). As seen in Figure 5a, while the addition of the A120 mAb clearly inhibited the generation of syncytia by R5 HIV-1_JR-FL _and p24 production, there was no detectable inhibition with the addition of LPS. The facts that LPS at 0.1 μg/ml failed to inhibit HIV-1 production (unlike the A120 mAb) and that the addition of anti-CD14 mAb (which blocks LPS binding to its receptor, CD14) did not reverse the inhibition of R5 HIV-1 infection suggest that the activity of the A120 mAb is not due to LPS contamination.

Altogether, these data document that the anti-human CXCR4 mAb, clone A120, which ligates CXCR4 molecules via the ECL1/ECL2 domains potently inhibited not only X4 but also R5 HIV-1 strains in freshly *in vitro *activated primary PBMC cultures.

### Enhancement of the production of the CCR5 binding β-chemokines and reduction of CCR5 expression by A120 mAb treatment

**Figure 6:**
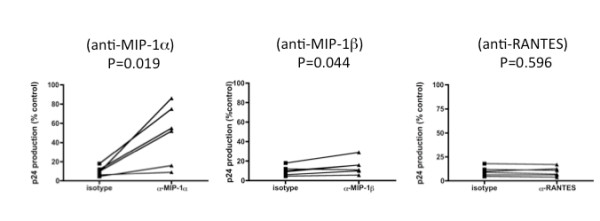
**Reversal of the A120 mAb-mediated inhibition of R5 HIV-1 infection in activated PBMCs with anti-CCR5 ligand-neutralizing mAbs**. Activated PBMCs from 6 donors were infected with R5 HIV-1_JR-FL _and cultured in the presence of 10 μg/ml A120 mAb or isotype control mAb together or without anti-chemokine mAbs including anti-MIP-1α, anti-MIP-1β or anti-RANTES at 10 μg/ml for 4 days. Virus production in the culture supernatants was determined by p24 ELISA. The p24 levels were plotted as percent of control values obtained in cultures incubated in each anti-β-chemokine mAb for each donor.

The present observations that the anti-CXCR4 A120 mAb inhibited the production of R5 HIV-1 in *a*ctivated PBMCs prompted us to examine whether CCR5 binding β-chemokines were involved. Thus, we tested whether neutralizing mAbs against human MIP-1α, MIP-1β and RANTES could reverse the effects of the A120 mAb on virus infection. As shown in Figure 6, indeed the A120 mAb-mediated inhibition of R5 HIV-1 infection was significantly reversed by anti-MIP-1α mAb and partially by anti-MIP-1β but not anti-RANTES mAb. These data suggest that MIP-1α and possibly MIP-1β were likely the major factors involved in the inhibition of R5 HIV-1 infection. As expected, the addition of these anti-β-chemokine mAbs did not reverse A120 mAb-mediated blocking of X4 HIV-1 infection (data not shown). However, this β-chemokine dependent mechanism for the inhibition of R5 HIV-1 by the addition of the A120 mAb is donor-dependent. Notably, the addition of the anti-chemokine mAbs failed to reverse the A120 mAb mediated inhibition of R5 HIV-1 in cultures of PBMCs from 2 of the 6 donors. The reason(s) for this resistance in these donors remains to be studied.

**Figure 7:**
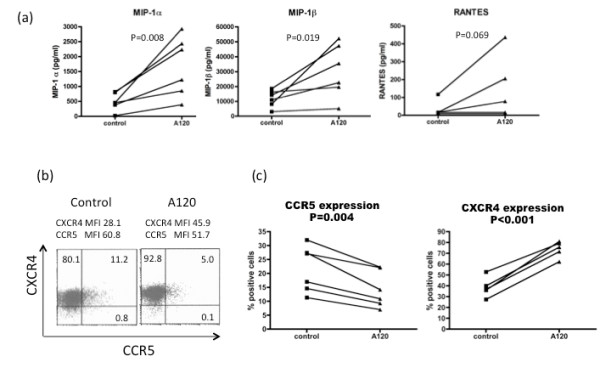
**The A120 mAb-treatment induces the production of CCR5 binding β-chemokines and the down-modulation of CCR5 expression**. PBMCs from 6 donors were activated with anti-CD3/28 mAbs for 1 day, washed, aliquoted and then incubated in the presence of 10 μg/ml A120 mAb or isotype control mAb for an additional day. (a) Changes in the concentrations of MIP-1α, MIP-1β and RANTES in the culture supernatants were assayed by ELISA. (b and c) Cells were analyzed for changes in the cell surface expression of CCR5 and CXCR4 on gated populations of CD4^+^T cells (MFI denotes mean fluorescence intensity). A representative flow cytometry dot blot profile is shown.

To confirm that the β-chemokines were indeed produced by the ligation of CXCR4 by the A120 mAb in activated PBMCs, we quantitated the concentration of these chemokines. Figure 7a shows that the A120 mAb enhanced the synthesis of MIP-1α and MIP-1β in most if not all the cases. Although enhanced RANTES production was seen in 3 out of the 6 donors, it is unlikely that RANTES is involved in the A120 mAb-mediated R5 HIV-1 inhibition as shown in Figure 6. As expected, treatment of activated PBMCs with the A120 mAb led to a significant reduction in the frequency of cells expressing CCR5 (Figure 7b and 7c). In contrast, there appeared to be a slight increase in the frequency of CXCR4 expressing CD4^+ ^T cells (Figure 7b and 7c). Therefore, these results indicate that the incubation of activated PBMCs in the presence of the A120 mAb inhibited R5 HIV-1 infection primarily via the blockade of the co-receptor function of CCR5, most likely due to its ability to induce the synthesis of CCR5-binding β-chemokines. It is important to note that the levels of MIP-1α induced by the A120 mAb showed a typical dose response curve (Additional file 1), and the level of R5 HIV-1 inhibition was inversely-correlated with levels of MIP-1α detected.

### Cell populations that produce the β-chemoknes

**Figure 8:**
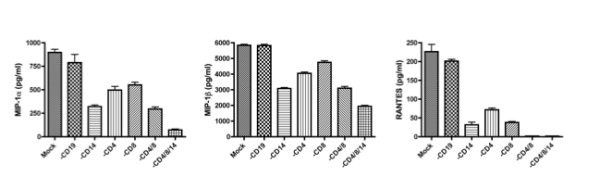
**The A120 mAb stimulates T cells and monocytes to produce β-chemokines in activated PBMCs**. One day-activated PBMCs were depleted of CD19^+^B cells, CD4^+^T cells, CD8^+^T cells and/or CD14^+^monocytes using immunobeads conjugated with appropriate lineage specific mAbs, and then cultured in the presence of A120 mAb or isotype control mAb for one day. Concentrations of MIP-1α, MIP-1β and RANTES in the culture supernatants were assayed by ELISA. Representative data from three independent experiments are shown.

In an effort to identify the cell lineage that was involved in the synthesis of the β-chemokines following incubation of the activated PBMCs in the presence of the A120 mAb, cell depletion experiments were carried out. Thus, aliquots of activated PBMCs were depleted of CD19^+ ^B cells, CD4^+ ^T cells, CD8^+ ^T cells or CD14^+ ^monocytes utilizing immune-beads conjugated with the appropriate lineage specific mAbs. Non-depleted (mock) and each cell lineage depleted PBMCs were cultured for 24 hours in the presence or absence of 10 μg/ml of A120 mAb. As shown in Figure 8, the most marked reduction in β-chemokine levels in the culture supernatants was noted in cultures depleted of CD14^+ ^monocytes followed by those depleted of CD4^+ ^T cells and CD8^+ ^T cells. However, B-cell depletion had minimal if any effect on the levels of β-chemokines synthesized. These results suggest that activated T cells along with monocytes were responding to the A120 mAb by secreting β-chemokines.

### A120 mAb exhibits broad HIV-1 clade inhibition

Finally, the unique availability of a panel of HIV-1 with distinct co-receptor usage and clades prompted us to examine the breadth of inhibitory activity of the A120 mAb. Once again, aliquots of 1-day anti-CD3/28 activated PBMCs were infected with 15 different HIV-1 strains and then cultured in the presence of 10 μg/ml of the A120 mAb or control IgG, and the levels of p24 in the supernatant fluids were quantitated on day 5 after infection. As shown in Table 1, incubation of the cultures in the presence of the A120 mAb uniformly led to a marked decrease in the levels of p24 for all ten R5 HIV-1 strains, three X4 HIV-1 strains, and two dual R5/X4 tropic HIV-1 strains (p = 0.0065).

**Table 1:** Suppressive effect of the A120 mAb on various clades of HIV-1 strains.

Member	HIV-1 Subtype	Isolate	Country of Origin	Syncytium	Co-receptor Usage	Percent inhibition of p24 production
PRD320-01	A	UG275	Uganda	NSI	CCR5	88.3%

PRD320-02	A	I-2496	Ghana	NSI	CCR5	99.8%

PRD320-03	CRF02_AG	DJ263	Djibouti	NSI	CCR5	94.7%

PRD320-04	CRF02_AG	POC44951	Liberia	NSI	CCR5	99.7%

PRD320-06	B	BZ167	Brazil	SI	CXCR4	97.2%

PRD3200-7	C	DJ259	Djibouti	NSI	CCR5	91.5%

PRD320-08	C	ZAM18	Zambia	NSI	CCR5	93.7%

PRD320-09	D	SE365	Senegal	SI	CXCR4	98.5%

PRD320-10	D	UG270	Uganda	SI	CXCR4	99.7%

PRD320-11	CRF01_AE	ID17	Indonesia	NSI	CCR5	81.0%

PRD320-12	CRF01_AE	NP03	Thailand	SI	CXCR4	94.5%

PRD320-14	F	BCI-R07	Romania	SI	CXCR4/CCR5	99.4%

PRD320-15	G	BCF-DIOUM	Zaire	NSI	CCR5	99.9%

PRD320-16	G	HH8793	Kenya	NSI	CCR5	83.3%

PRD320-17	H	BCF-KITA	Zaire	NSI	CCR5	92.5%

PRD320-18	O	BCF06	Cameroon	SI	CXCR4/CCR5	98.3%

PRD320-19	O	I-2478B	US	NSI	CCR5	65.6%

Anti-CD3/CD28 activated PBMCs were infected with each of 15 different HIV-1 strains belonging to various clades and with previously defined different CXCR4 and CCR5 usages. HIV-1 dose of 10 ng p24 value was added to 1 × 10^6 ^cells for infection. After washing, PBMCs were aliquoted and cultured in triplicate in the presence of 10 μg/ml of the A120 mAb or isotype control IgG for 5 days. Virus production was determined by quantitation of p24 in the culture supernatants by ELISA and the mean values calculated. Percent inhibition was calculated relative to the values obtained with the isotype control mAb alone. Representative data from three independent experiments are shown.

## Discussion

The present study is the first report that documents the unique property of an anti-humanCXCR4 mAb (clone A120) which upon ligation of CXCR4 via the ECL1/ECL2 domains strongly blocks the infection of not only X4 but also R5 and dual tropic HIV-1 strains in freshly *in vitro *activated PBMC cultures. The mechanism for the inhibition of the X4 HIV-1 is likely due to direct interference and binding of gp120 to CXCR4 as reported previously. In addition, since A120 mAb treatment increases CXCR4 expression on CD4^+ ^T cells (Figure 7), it may also be possible that the A120 mAb may block X4 HIV-1 infection by interfering with CXCR4 trafficking. By contrast, the predominant mechanism for the inhibition of the R5 HIV-1 infection by the A120 mAb is most likely due to the production of the CCR5-binding β-chemokines, especially MIP-1α, from activated T cells and monocytes leading to down-modulation of CCR5 expression on CD4^+ ^T cells. The observations that the anti-CXCR4 N-terminus mAb (clone A145) showed little or no inhibition, and the anti-CXCR4 ECL3 mAb (clone A80) was not as potent in inhibiting HIV-1 infection, as compared with the A120 mAb, indicate that the ligation via the ECL1 and/or ECL2 domains is critical for the inhibition of R5 and X4 HIV-1 infection. This view is supported by the finding that a panel of commercially available murine mAbs, whose reactive sites were localized to the ECL1/ECL2 domains or the single ECL2 domain of CXCR4, also showed similar, but less effective suppressive effects on infection with both the X4 and R5 HIV-1 and enhanced MIP-1α and β production under the same culture conditions presented herein (data not shown).

Preliminary data indicate that chemically inactivated X4 HIV-1 (HIV-1IIIB) and recombinant SDF-1 did not induce the synthesis of such β-chemokines or inhibit R5 HIV-1 infection in activated PBMCs (data not shown). Thus, it is important to point out that ligation of CXCR4 by its natural ligand SDF-1 or HIV-1 gp120 is not sufficient for generating signals suitable for the synthesis of the CCR5 ligands, and that ligation of CXCR4 via specific domains is required for these unique anti-HIV-1 activities.

So far, similar suppression of both X4 and R5 HIV-1 infection has also been reported in a study utilizing anti-human CCR2 mAb that is neither agonistic nor antagonistic [14]. It was reasoned that this anti-CCR2 mAb functions by the induction of hetero-oligomerization of CCR2 with CCR5 and CXCR4, but not receptor down-modulation. Another report showed that a non-agonistic/antagonistic anti-CCR5 N-terminus specific mAb that is unable to block the binding of R5 HIV-1 gp120 to CCR5 interferes with R5 HIV-1 infection by induction of CCR5 dimerization rather than down-modulation of CCR5 [16]. It is of interest to note that this anti-CCR5 mAb does not inhibit X4 HIV-1. Thus, our finding that ligation of CXCR4 via the ECL1/ECL2 region on activated PBMCs results in the production of CCR5-binding β-chemokines followed by down-modulation of CCR5 expression is unique. However, it remains to be determined whether the ligation of CXCR4 with the A120 mAb similarly induces hetero-dimerization of CXCR4 with CCR5 or the other chemokine receptors or CCR5 homo-dimerization. Further studies are in progress using immunoprecipitation and Western blot techniques utilizing appropriate mAbs.

It is important to note that the addition of anti-chemokine mAbs did not show the same degree of reversal of the A120 mAb-induced inhibition of R5 HIV-1 infection in the cultures from 2 out of the 6 PBMC donors (Figure 6). In addition, there was a lack of correlation between enhanced β-chemokine levels and the reversing effects of the anti-β-chemokine antibodies on the A120-mediated R5 HIV-1 inhibition. We assume that the concentration of the β-chemokine antibodies (10 μg/ml) was sufficient to neutralize endogenously produced β-chemokines as the antibodies at this concentration could neutralize > 100 ng/ml of each of the recombinant β-chemokines (data not shown). While resistance of these donors was not due to the production of some other anti-HIV-1 factor such as CD8^+^T lymphocyte antiviral factor (CAF) [17], it may be possible that treatment with the A120 mAb might induce the hetero-dimerization of CXCR4 and CCR5 which results in resistance to R5 HIV-1 infection. Further studies are in progress to address this issue. It is interesting to note that among the neutralizing mAbs against the β-chemokines, the anti-MIP-1α mAb was the most effective in reversing the A120 mAb-induced R5 HIV-1 inhibition. Since all the available anti-MIP-1α mAbs at present do not distinguish MIP-1α (LD78α) from its homologue CCR3L1 product (LD78β) [18], it is possible that CCR3L1 protein is also produced upon A120 mAb treatment and involved in the R5 HIV-1 inhibition. As CCR3L1 is known to be a potent factor that may delay the progression to clinical AIDS [19], it will be important to determine whether A120 mAb stimulates the production of CCR3L1 proteins. Such studies are also in progress.

The generation of resistance to CCR5 inhibitors involving either the selection of pre-existing CXCR4 tropic HIV-1 and/or due to the evolution of Env variants has been well documented [20]. Thus, in such cases, the availability of a reagent like the A120 mAb that has inhibitory properties for both CCR5 and CXCR4 tropic HIV-1 may provide a unique therapeutic tool worthy of consideration. Since the A120 mAb also inhibits the SIV-1 infection in activated PBMCs from rhesus macaques (Takahashi et al., unpublished), this hypothesis is currently being investigated using the nonhuman primate model.

## Conclusions

Data described herein have identified a unique epitope of CXCR4 whose ligation not only directly inhibits CXCR4 tropic HIV-1, but also indirectly inhibits the infection of R5 tropic HIV-1 via the synthesis of natural CCR5 ligands.

## Methods

### Reagents

RPMI 1640 medium (Sigma-Aldrich. Inc. St. Louis, MO) supplemented with 10% fetal calf serum (FCS), 100 U/ml of penicillin and 100 μg/ml of streptomycin (hereinafter called RPMI medium) was utilized for the described studies. Anti-human CD3 (clone OKT-3) and anti-CD28 (clone 28.2) were obtained from the American Type Culture Collection (Rockville, MD) and BioLegend (San Diego, CA), respectively. Neutralizing mAbs against human RANTES, MIP-1α and MIP-1β were purchased from R&D systems (Minneapolis, MN). The rat anti-CXCR4 mAbs used were produced in our laboratory and included clones A145 (IgG1), A120 (IgG2b) and A80 (IgG1) [16]. Mapping of the epitopes recognized by these mAbs was reported previously [16]. Other rat mAbs used were IgG1 anti-CCR5, IgG2b anti-HTLV-I gp46 and IgG1 anti-HCV produced in our laboratory [16,21,22]. These mAbs were purified from CB.17-SCID mouse ascites fluids by ammonium sulfate precipitation followed by gel filtration using Superdex G-200 (GE), and passed through a polymyxin B column to remove potential LPS contamination. The fluorescent dye-labeled anti-human CD4, CD8, CD14 and CD19 mAbs were purchased from Beckman-Coulter or BioLegend. The anti-HIV-1 p24 mAbs used were also produced in our laboratory. Magnetic beads conjugated with mAbs against human CD4, CD8, CD14 or CD19 were purchased from Dynal and used according to the manufacturer's recommendation. Low endotoxin murine anti-CXCR4 mAbs including clone 12G5 and the other anti-CXCR4 ECL2 mAbs were purchased from BioLegend and R&D.

### HIV-1 preparation

Virus stocks of R5 HIV-1_JR-FL_, R5 HIV-1_JR-CSF _and X4 HIV-1_NL4-3 _were produced by transfection of the 293T cells with the appropriate HIV-1 infectious plasmid DNAs utilizing the calcium phosphate method as described previously [23]. X4 HIV-1_IIIB _was produced in the Molt-4/IIIB cell line. The other HIV-1 isolates used were from the HIV subtype infectivity panel PRD320 (BBI Diagnostics, West Bridgewater, MA, USA) which included clade A R5 HIV-1 (UG275, I-2496 isolates), clade CRF02AG R5 HIV-1 (DJ263, POC44951 isolates), clade B R5 (US2 isolate) and X4 HIV-1 (BZ167 isolate), clade C R5 HIV-1 (DJ259, ZAM18 isolates), clade D X4 HIV-1 (SE365, UG270 isolates), clade CRF01AE R5 (ID17 isolate) and X4 HIV-1 (NP03 isolate), clade F R5 (BZ163 isolate) and X4/R5 HIV-1 (BCI-R17 isolate), clade G R5 HIV-1 (BCF-DIOUM, HH8793 isolates), clade H R5 HIV-1 (BCF-KITA isolate), clade O R5 (I-2478B isolate) and X4/R5 HIV-1(BCF06 isolate). Each of these panel HIV-1 strains was grown in primary PHA-activated PBMCs and the levels of p24 determined and 10 ng of p24 used to infect PBMCs. These HIV-1 stocks were aliquoted and stored at -80°C until used.

### In vitro stimulation of PBMCs and infection with HIV-1

PBMCs from healthy donors were obtained by density gradient centrifugation on HistoPAQUE-1077 (Sigma-Aldrich), suspended at 2 × 10^6 ^cells/ml in RPMI medium, dispensed into individual wells of 24-well plates (BD) (1 ml/well) pre-coated with 5 μg/ml anti-CD3 mAb (OKT-3) and cultured in the presence of soluble 0.1 μg/ml anti-CD28 mAb at 37°C in a 5% CO_2 _humidified atmosphere for 24 hours. The activated PBMCs were collected, washed once and infected with HIV-1 at a multiplicity of infection (m.o.i.) of 0.005~0.01 or at 10 ng p24 per 1~2 × 10^6 ^cells for 2 hours. Infected PBMCs were washed three times, re-suspended at 0.5~1 × 10^6 ^cells/ml in RPMI medium containing 20 U/ml recombinant human IL-2 containing RPMI medium, dispensed into individual wells of 48-well plates (BD) (0.5 ml/well) and then cultured in the presence or absence of various concentrations of the anti-CXCR4 or control mAbs. Production of HIV-1 was determined by the measurement of HIV-1 core p24 levels by ELISA, and the number of HIV-1 p24^+ ^cells were determined by FCM as described previously [24]. For select experiments, activated PBMCs were cultured at 1 × 10^6 ^cells/ml in RPMI medium containing 20 U/ml IL-2 in the presence or absence of 10 μg/ml of A120 mAb for 24 hours, and the culture supernatants were collected, and the levels of β-chemokines were determined by ELISA. All the experiments in this study were performed in triplicate wells.

### Cell lines

Molt-4/IIIB [25] and MT-2 [26] cells that were productively infected with HIV-1IIIB (Molt-4/IIIB) and human T cell leukemia virus type-I (HTLV-I), respectively, were cultured in RPMI medium. HIV-1 and HTLV-I production were determined by our in-house HIV-1 p24 and HTLV-I p24 sandwich ELISA kits (Tanaka et al., unpublished).

### Flow Cytometry (FCM)

Cells to be analyzed were Fc-blocked with 2 mg/ml normal human pooled IgG on ice for 15 minutes, and aliquots of these cells were subjected to staining using pre-determined optimum concentrations of fluorescent dye-conjugated mAbs for 30 minutes on ice. The cells were then washed using FACS buffer (PBS containing 2% FCS and 0.1% sodium azide), fixed in 1% paraformaldehyde (PFA) in FACS buffer and analyzed using a FACS Calibur. The data obtained were analyzed using the Cell Quest software (BD). For detection of HIV-1 infected cells, cells were fixed with 4% PFA-containing PBS for 5 min at room temperature followed by washing with 0.1% Saponin-containing FACS buffer. These cells were then Fc-blocked with 2 mg/ml normal human pooled IgG on ice for 15 min, and aliquots of these cells were stained with Alexa Fluor 488-conjugated anti-HIV-1 p24 mAb (clone 2C2) for 30 min on ice. The cells were then washed using FACS buffer and the frequency and the absolute number of p24+ cells determined by FCM using a cell counting kit (BD) according to the manufacturer's protocol.

### Statistical analysis

Data were tested for significance using the Student's *t *test using the Prism software (GraphPad Software).

## Lists of abbreviations used

HIV: human immunodeficiency virus; PBMC: peripheral blood mononuclear cells; mAb: monoclonal antibody; X4: CXCR4-tropic; R5: CCR5-tropic; ECL: extra-cellular loop.

## Competing interests

The authors declare that they have no competing interests.

## Authors' contributions

TA and RT performed research, analyzed data, and wrote the manuscript. AK, SM, and Takahashi contributed to experiments and analyzed data. AAA contributed to designing research and wrote the manuscript. YT designed and preformed research, wrote the manuscript and provided funding for this study. All authors read and approved the final manuscript.

## Supplementary Material

Additional file 1 **Dose response of the A120 mAb-mediated MIP-1α production in activated PBMCs**. As described in the legend for Figure 7, activated PBMCs were incubated in the presence of graded concentrations of the A120 mAb or isotype control mAb for an additional day. Changes in the concentrations of MIP-1α in the culture supernatants were assayed by ELISA. Isotype control mAbs did not enhance MIP-1α production at 0.5~20 μg/ml in these culture conditions (data not shown). Representative data are from 3 independent experiments using PBMCs from a single donor. Click here for file
