# Supplementary figures and images for: Identification of an unique CXCR4 epitope whose ligation inhibits infection by both CXCR4 and CCR5 tropic human immunodeficiency type-I viruses
Source: Retrovirology. 2011 Oct 22;8:84. doi: 10.1186/1742-4690-8-84 (PMC3239297; doi:10.1186/1742-4690-8-84)

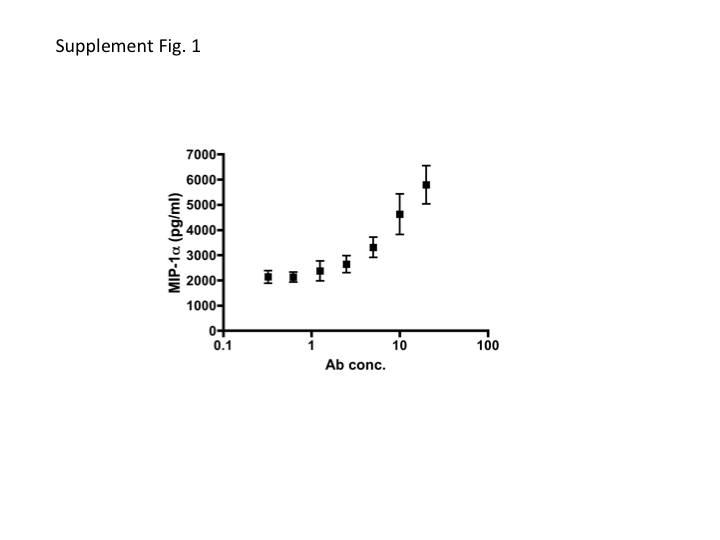

Supplement: Additional file 1 — Dose response of the A120 mAb-mediated MIP-1α production in activated PBMCs. As described in the legend for Figure 7, activated PBMCs were incubated in the presence of graded concentrations of the A120 mAb or isotype control mAb for an additional day. Changes in the concentrations of MIP-1α in the culture supernatants were assayed by ELISA. Isotype control mAbs did not enhance MIP-1α production at 0.5~20 μg/ml in these culture conditions (data not shown). Representative data are from 3 independent experiments using PBMCs from a single donor. [file 1742-4690-8-84-S1.TIFF]
